# Effects of concentrated urine on complications and plasma creatinine: a prospective study in elective non-cardiac surgery patients

**DOI:** 10.3389/fmed.2025.1662177

**Published:** 2025-11-17

**Authors:** Kai Xie, Yuhong Li, Wenjie Zou, Rui He, Robert G. Hahn

**Affiliations:** 1 Department of Anesthesiology, Shaoxing People’s Hospital, Zhejiang University, Shaoxing, China; 2 Department of Anesthesiology, Shulan (Hangzhou) Hospital, Shulan International Medical College, Zhejiang Shuren University, Hangzhou, China; 3 Department of Clinical Medicine, Wannan Medical College, Wuhu, China; 4 Karolinska Institutet at Danderyds Hospital (KIDS), Stockholm, Sweden

**Keywords:** acute kidney injury, surgery, blood creatinine, hydroxyethyl starch, hypotension, postoperative complications, urine creatinine

## Abstract

**Objectives:**

The urine concentration of metabolic end products increases in response to low habitual water intake or acute dehydration. We examined the impact of concentrated urine on surgical outcomes with special attention given to plasma creatinine levels.

**Methods:**

A prospective observational study was conducted involving 921 patients scheduled for non-cardiac major surgery. The degree of urine concentration was quantified prior to the surgery using a composite index (Fluid Retention Index, FRI) that reflects renal water conservation. Arterial pressure was monitored every 5 min during the operations. A perioperative increase in plasma creatinine of >50% or ≥26.5 μmol/L was designated as acute kidney injury (AKI).

**Results:**

The average operating time averaged 2.9 ± 1.3 h (mean ± SD) during which the mean arterial pressure was 5.2 ± 13.1 mmHg lower than the preoperative reading. Concentrated urine (FRI > 4.0) was present in just 7% of the patients, signifying that dehydration was infrequent. Univariate analysis showed that these patients still had extended gastrointestinal recovery time (*p* < 0.001), larger hemorrhages (5% vs. 1% > 500 mL; *p* = 0.047), and a heightened occurrence of fever (28% vs. 17%; *p* < 0.03). Multivariate analysis showed an extended gastrointestinal recovery time and smaller urine output despite receiving more crystalloid fluid (all correlations *p* < 0.001). Gradually higher FRI was associated with lower MAPs at baseline (*p* < 0.024). Postoperative AKI developed in only 1% of the patients, which made the study underpowered to detect a statistically significant relationship between concentrated urine and AKI (odds ratio 0.988, 95% confidence interval 0.980–0.996; *p* = 0.43).

**Conclusion:**

Patients with concentrated urine before surgery had a lower urine output during surgery and a longer postoperative period of food intolerance and more often fever than patients with dilute urine. The occurrence of postoperative AKI was very low, which was probably due to the generally good hydration status.

## 1 Introduction

Postoperative complications are costly in terms of suffering and economy, but preventive measures require good understanding of the conditions that promote their development. “Concentrated urine” is rarely considered to influence the incidence of surgical complications. This trait indicates that the urinary concentrations of metabolic end products are high because of renal water conservation, which might be due to acute dehydration but, more commonly, to low daily water consumption (1–3). Only a few small studies suggest that concentrated urine impacts surgical complications. For example, 60% of those with concentrated urine who underwent hip fracture surgery had at least one postoperative complication, while the incidence was 30% in the others.

Acute kidney injury (AKI) is a postoperative complication of special interest as concentrated urine brings the patient closer to the renal threshold for creatinine excretion (4). In a study of 642 surgical patients from four countries, concentrated urine before surgery was associated with low urine flow and increased incidents of postoperative AKI (5). This clinical syndrome is common after major surgery (usually 10%) and associated with higher long-term morbidity and mortality (6). AKI denotes an abrupt decrease of kidney function that might be due to specific kidney diseases and non-specific conditions such as ischemia and toxic injury, and well as to extrarenal pathology (7). The diagnosis is based on an acute elevation of plasma creatinine (+50% or ≥26.5 μmol/L) and/or on persistent oliguria (7, 8). Plasma creatinine typically hits its highest point on the first or second postoperative day, whereafter normalization usually (but not always) occurs (9). Aside from plasma creatinine, no sufficiently powered study has yet compared the effects of concentrated urine on the course of anesthesia and surgery.

The aim of the present study was to evaluate the relationship between concentrated urine measured prior to non-cardiac elective surgery and postoperative complications, with a particular focus on plasma creatinine. Concentrated urine was assessed using the Fluid Retention Index (FRI) scale, which is a summary measure of four biomarkers that have been used to quantify renal water conservation in dietary studies (2, 3), sports medicine (10–12), and surgery (4, 5, 13). The robust FRI scale integrates these biomarkers into a single value, which is an approach so far employed in approximately 15 studies in geriatric care and surgery (14–20).

The study hypothesis was that concentrated urine poses a risk of postoperative morbidity, including the development of AKI.

## 2 Methods

### 2.1 Study design and setting

We conducted a single-center, prospective clinical study at Shaoxing People’s Hospital, which is university hospital in the People’s Republic of China.

### 2.2 Patients

The study included 921 patients who underwent elective open and laparoscopic surgeries, the majority of which was performed due to a cancer diagnosis. The criteria for enrolment included being in the ASA Class I–II, aged between 18 and 100 years, and having a body mass index of 18–30 kg/m^2^. Those with urogenital diseases or severe cardiac, lung, renal, or hepatic disease were excluded. Hence, patients with pre-existing kidney disease were excluded. The types of studied surgeries are listed in Supplementary File 1. Patients were recruited during two periods, from June 2018 and 1 year forward, and between February and June, 2023. The first 126 patients have been reported previously (20) after which the included measurements were adopted as a routine. This presentation follows the STROBE checklist.

### 2.3 Procedures

The patients did not ingest solid food from midnight. They arrived at the operating theatre between 7 a.m. and 9 a.m. None were given premedication. General anesthesia was induced with midazolam 50 μg/kg, propofol 1.5 mg/kg, cis-atracurium 0.15 mg/kg, and sufentanil 5 μg/kg. This was followed by endotracheal intubation and mechanical ventilation. The anesthesia was maintained with propofol 6 mg/kg/h and 1.5 MAC of sevoflurane. Additional cis-atracurium 0.05 mg/kg and sufentanil 2 μg/kg was given as required.

Intravenous (i.v.) fluid therapy consisted in lactated Ringer’s solution (Pharmacia-Baxter, Shanghai, China) and 6% hydroxyethyl starch 130/0.4 (Voluven^®^; Fresenius Kabi Deutschland GmbH, Bad Homburg, Germany) but were not given according to a strict protocol. Voluven^®^ was initiated during the induction of anesthesia with the purpose of maintaining stable hemodynamics. Blood products were given at the clinicians’ discretion.

### 2.4 Data collection

Monitoring consisted of pulse oximetry, heart rate, invasive arterial pressure, and electrocardiography. A catheter was placed in the radial artery before induction of anesthesia and the mean arterial pressure (MAP) recorded every 5 min from the preoperative phase until the end of the surgery. The data was stored on a DoCare Anesthesia Clinical Information System (Medical System, Shuzhou, China).

The “patient mean MAP” refers to the mean of the MAP measurements in a single patient but reported separately for measurements made before and after anesthesia was induced. We identified instances of hypotension (MAP < 60 mmHg) and noted the total duration of hypotension in each patient. According to a systematic review 60 mmHg is the most frequently used threshold for hypotension during surgery (21). Ephedrine 5–10 mg was given i.v. if a patient experienced both hypotension and bradycardia. Continuous infusions of catecholamines were not used.

Blood and urine samples were collected prior to anesthesia induction. Additional blood samples were gathered on the first postoperative day, and for 96% of the patients up to 9 days post-surgery. These samples were evaluated for plasma creatinine and C-reactive protein levels in the hospital’s Clinical Chemistry Laboratory. Additional blood analyses, including protein, albumin, liver enzymes, and immunoglobulins, are available in Supplementary File 2.

Blood loss was determined by measuring the blood volume in the suction tubes and weighing the sponges. Urine volume was measured and sampled from the start of anesthesia until discharge from the postoperative care unit, using an indwelling catheter. The first portion of the collected urine was tested for color, gravity, osmolality, and creatinine. Color was assessed using a UrineHydration chart (en.wikipedia.org/wiki/File:Urine_Hydration.chart.jpg) (1).

### 2.5 Fluid Retention Index (FRI)

The Fluid Retention Index (FRI) was used to quantify the urine concentration before the surgeries. This scale was constructed in 2013 and has been applied in approximately 15 studies in sports medicine, surgery, and geriatric care. The FRI is a composite index of renal water conservation based on four urinary biomarkers: urine-specific gravity, creatinine, osmolality, and urine color. These variables represent metabolic waste products that are excreted at a constant rate regardless of variations in urine flow.

The FRI concept is based on the curvilinear relationship between urine-specific gravity and the change in body weight found during progressive dehydration in 7 published studies in sports medicine and further validated in volunteers performing recreational sports activities (12). Urine-specific gravity, urine osmolality, and urine creatinine also distinguish between volunteers who have different daily consumptions of water (2, 3). There are strong inter-correlations between the four biomarkers; correlation coefficients range from 0.71 to 0.84 (12), and this correlation strength was consistent when re-tested with 300 hospital workers (1).

The FRI scale is constructed so that each of the four biomarkers is assigned a score ranging from 1 to 6, with the average score representing the final FRI value (Table 1). The boundaries for the FRI scores were decided based on the relationships between the four indices of dehydration as obtained in 57 subjects aged 17–69 years (12) and in 256 patients admitted to hospital for acute geriatric care (14). Despite the strong correlations, using a composite index provides less sensitivity to outliers than individual biomarkers, which can occasionally be affected by diet and medication.

**Table 1:** Description of the four dimensions of the Fluid Retention Index (FRI).

Score	1	2	3	4	5	6
Specific gravity	≤1.005	1.010	1.015	1.020	1.025	1.030
Osmolality (mOsmol kg^−1^)	<250	250–450	450–600	600–800	800–1,000	>1,000
Creatinine (mmol L^−1^)	<4	4–7	7–12	12–17	17–25	>25
Color (shade)	1	2	3	4	5	6

FRI > 4.0 indicates a 3% body weight reduction due to dehydration after physical activity (10–12). This cut-off was used to differentiate between properly hydrated patients and those who are dehydrated.

Patients were not managed in any particular way to account for their preoperative hydration status as the result was not known to the anesthesiology team.

### 2.6 Outcome measures

Data on postoperative complications (other than AKI) were extracted from the medical records and thus represent those routinely tracked by the hospital. These complications included respiratory infection, would infection, fever, reoperation, pain, need for intensive care, and length or hospital stay. Pain was quantified using a visual analogue scale graded from 0 to 10 where 10 was the worst possible pain intensity; the number of occasions where a patient scored >4.0 were counted. The definitions used for the International Surgical Outcomes Study (ISOS, see http://isos.org.uk) were used. We also recorded the gastrointestinal recovery time (i.e., end of paralytic ileus determined by the return of bowel sounds) and when the oral food intolerance time ended.

### 2.7 Acute kidney injury

Acute kidney injury (AKI) was diagnosed based on an increase of the plasma creatinine concentration by ≥ 50% or an increase by ≥ 26.5 μmol/L on the first or second postoperative day (6–8). AKI can also be diagnosed based on low urine output (<0.5 mL/kg/h for >6 h) but was not used here as oliguria is less clearly associated with poor outcomes than AKI diagnosed by plasma creatinine (8).

### 2.8 Statistics

Data showing a normal distribution are presented as the mean ± standard deviation. Changes were studied by the paired *t* test. Differences between groups were assessed using one-way analysis of variance (ANOVA) followed by the Scheffé *post hoc* test (if >2 groups).

Data with a skewed distribution are presented as the median and the 25th–75th percentile, and differences were evaluated using the Mann–Whitney *U* test (for 2 groups) or the Kruskal-Wallis test (for ≥3 groups) followed by the pairwise *post hoc* test in SPSS version 30.0.0 for Mac (IBM Corp., Armonk, NY). The Hodges-Lehmann estimate was used to report the 95% confidence interval (CI) for the differences between the data in two groups where the distribution was skewed.

For categorical data, the chi-square test was used, with squared *z*-values determining statistical differences between sub-groups.

Univariate and stepwise multiple linear and logistic regression (Hosmer-Lemeshow goodness-of fit) were used to identify demographic and surgical variables that correlated significantly with the outcome measures. *p* < 0.05 was considered statistically significant.

The power analysis focused on determining if a high FRI score before surgery was statistically more common among patients who developed postoperative AKI. Our pooled data study from four countries revealed high FRI scores in 24% of the patients, and AKI developed in 6.1% (5). The required number of patients would then be 875, which provided 90% statistical power at the *p* < 0.01 significance level.

## 3 Results

### 3.1 Baseline data

The analysis involved 921 patients (47% females) with an average age of 61 ± 12 years, body weight of 60 ± 9 kg, and body mass index (BMI) of 22.8 ± 2.8 kg/m^2^. Of these patients, 9% were over 75 years old, and 19% had a BMI greater than 25 kg/m^2^. Data were complete except for AKI which was available in 773 patients.

**Figure 1:**
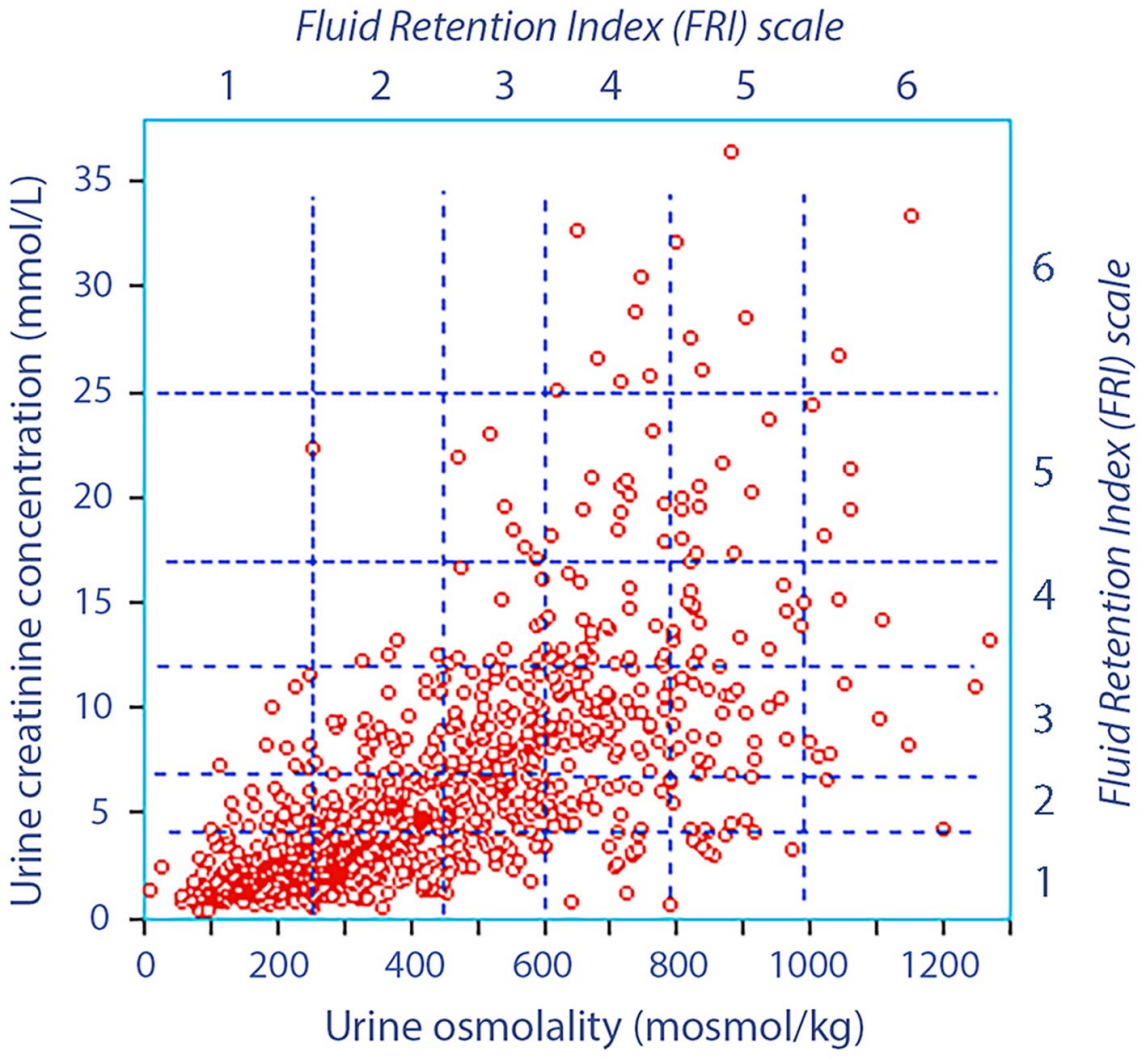
Urine osmolality *versus* the urine creatinine concentration before surgery in 921 patients. The vertical hatched lines indicate the FRI scores with respect to urine osmolality and the horizontal lines the divisions with respect to urine creatinine. Hence, each square gives one FRI score for urine creatinine and another score for urine creatinine, and the final FRI value is the mean of four scores (the other two being urine color and urine-specific gravity).

The average FRI was 2.3 ± 1.1, with only 64 patients (7%) scoring >4.0 as evidence of dehydration (Figure 1). The basic data classified by surgery type is presented in Supplementary Table S1.

### 3.2 Perioperative data

The surgery lasted for 2.9 ± 1.3 h, with 63% of the procedures carried out laparoscopically or thoracoscopically. The Ringer’s solution given amounted to 1,998 ± 724 mL and 93% of the patients received 500 mL of Voluven.

**Figure 2:**
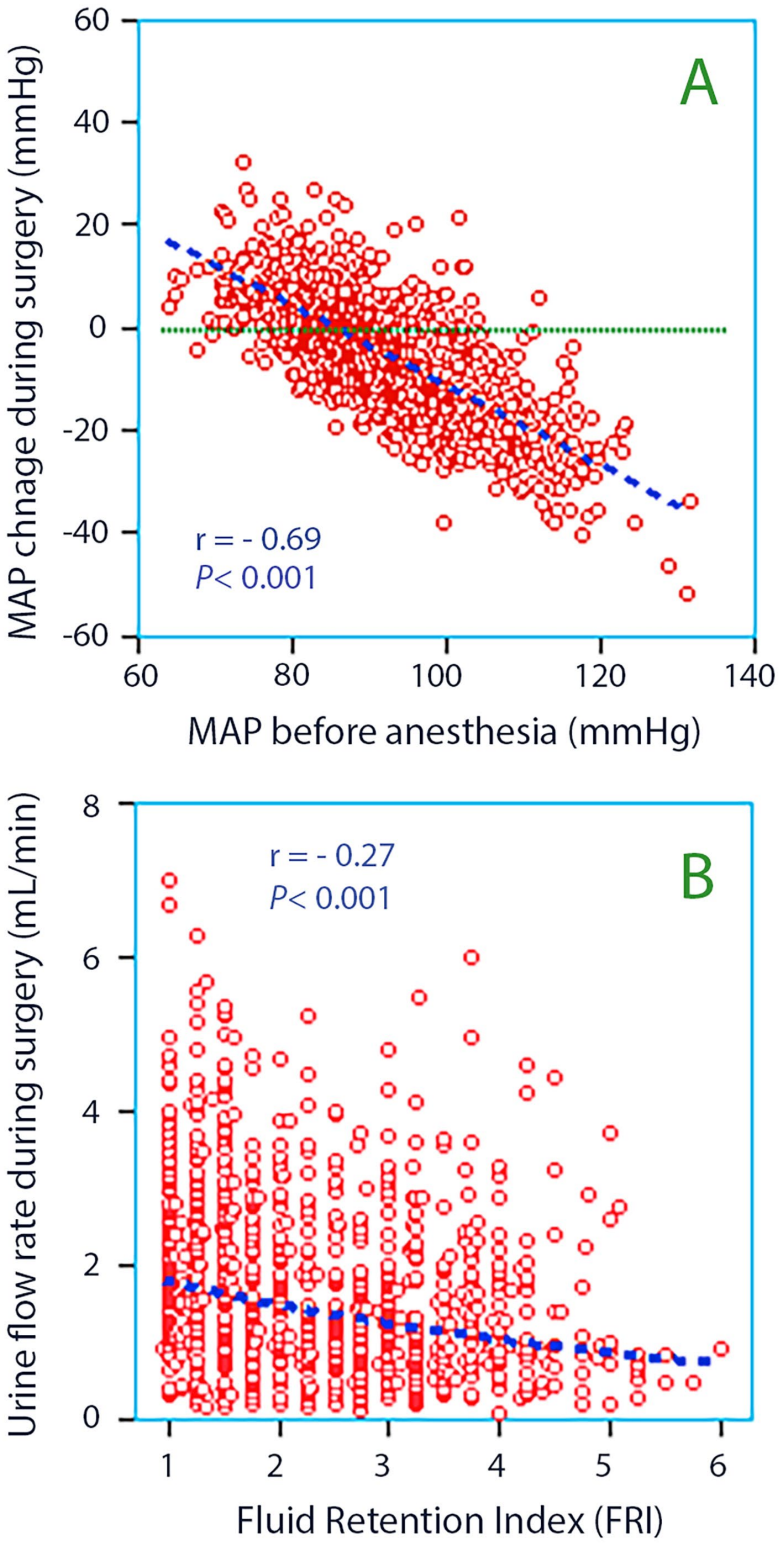
**(A)** The mean arterial pressure (MAP) measured before anesthesia was induced *versus* the change that occurred during anesthesia and surgery. The latter was obtained as the mean of all invasive measurements taken every 5 min throughout each surgery. Simple linear regression was used for the statistics. **(B)** Fluid Retention Index (FRI) *versus* the urine flow rate during surgery. Whether the kidneys are pre-set to retain or excrete fluid influences the urine flow.

The patient mean MAP during surgery (87 ± 10 mmHg) was only slightly lower than before the surgery (92 ± 10 mmHg: paired *t* test *p* < 0.001). The mean change was −5.2 ± 13.1 mmHg, the greatest reductions occurring in those having a high MAP at baseline (Figure 2A).

Hypotension (<60 mmHg) occurred in 122 patients (13.3%); one fifth (26 patients) showed more than two 5-min recordings of hypotension. MAP during hypotension was 54 ± 7 mmHg and the total hypotension time was 70 min of a total of 162,096 min (0.043%).

The median blood loss was 80 mL (25th–75th percentile limits, 50–148 mL), and the urine output was 350 mL (200–580), corresponding to a flow of 1.3 mL/min (0.8–2.1).

The plasma concentration of C-reactive protein was 1.2 μg/L (0.5–3.2) before the surgery and 51 μg/L (33–89) postoperatively. Blood products were given to 147 patients.

After the surgery, paralytic ileus persisted for 1.5 (0.3–2.0) days and the time of food intolerance was 2.0 days (0.3–4.0). Post-surgery, 18% of the patients experienced fever, 21% contracted a respiratory infection, and 10% developed a wound infection.

Ten patients were reoperated (1.1%). Intensive care was needed for 25 patients (2.7%). The length of the hospital stay for the patients needing intensive care was 14.0 ± 5.4 days while being 9.0 ± 4.2 days for the others (*p* < 0.001).

The outcomes in the sub-groups are displayed in Supplementary Table S2. No significant difference was observed in the perioperative change in plasma creatinine between them.

### 3.3 High FRI, univariate analyses

Patients with FRI > 4.0 were younger and received more Ringer’s solution but still had lower urinary flow than the other patients (Figure 2B). They were more often males, had lower MAP at baseline, experienced larger surgical hemorrhages, prolonged paralytic ileus, and food intolerance post-surgery. These patients also had a higher rate of fever and wound infection compared to patients with FRI ≤ 4.0 (Table 2). Hypotensive events were not more prevalent among those with high FRI; it was even associated with unchanged MAP during the surgical period relative to the preoperative records.

**Table 2:** Differences between patients having a Fluid Retention Index (FRI) of >4.0 on admission to hospital as evidence of strong renal water conservation before the surgery.

	FRI ≤ 4.0 (*N* = 857)	FRI > 4.0 (*N* = 64)	Odds ratio or difference (95% CI)	*P*-value
FRI (mean score)	2.1 ± 0.9	4.7 ± 0.5	2.58 (2.35–2.81)	<0.001
Age (yr)	61.3 ± 11.9	52.6 ± 12.6	8.92 (5.85–12.0)	<0.001
Females (%)	48	34	0.58 (0.34–0.98)	0.049
ASA I, II, III^*^	78/20/2	80/16/4	–	0.12
Infused ringer				
Volume (mL)	1,974 ± 708	2,320 ± 848	345 (161–529)	<0.001
>3 L (%)	5.7	14.3	2.75 (1.28–5.89)	0.007
Infused Voluven (mL)				
Incidence (%)	93%	94%	–	0.85
Volume (mL)	551 ± 177	717 ± 250	151 (75–228)	0.001
Urine (mL)	380 (200–600)	200 (118–500)	100 (50–170)	0.003
Urine flow (mL/min)	1.6 (0.9–2.5)	0.9 (0.6–1.7)	0.70 (0.23–1.21)	<0.001
Baseline (mmHg)	92 ± 12	89 ± 10	3.4 (0.4–6.4)	0.03
Surgery/baseline (ratio)	0.95 ± 0.14	1.01 ± 0.17	0.062 (0.39–1.05)	0.002
Blood loss				
Volume (mL)	80 (50–120)	100 (30–170)	42 (10–73)	0.01
>500 mL (%)	1	5	4.86 (1.28–18.43)	0.04
Extubation time (min)	27 (18–40)	30 (21–50)	5 (1–10)	0.001
P-glucose (mmol/L)				
Baseline	5.0 ± 0.6	5.1 ± 0.5	–	0.47
Postoperative	6.6 ± 1.8	5.8 ± 1.6	0.88 (0.33–1.43)	0.006
P-creatinine (μmol/L)				
Baseline	65.6 ± 13.7	63.5 ± 14.2	–	0.21
Change (%)	+0.7 ± 16.0	−4.4 ± 16.0	3.42 (0.61–6.24)	0.017
Paralytic ileus (days)	1.5 ± 1.3	2.4 ± 2.3	0.81 (0.46–1.16)	0.001
Food intolerance (days)	2.4 ± 2.3	3.9 ± 3.2	1.52 (0.70–2.35)	0.011
Infection (%)				
Respiratory	21	19	0.87 (0.46–1.67)	0.72
Wound	10	17	1.99 (1.00–3.96)	0.046
Fever	17	28	1.89 (1.07–3.35)	0.03

The statistical test was one-way ANOVA or the Mann–Whitney’s *U* test depending on data distribution. Categorical data were evaluated by the chi-square test. Bonferroni correction of the *p*-values to account for multiple testing was applied.

FRI, Fluid Retention Index; MAP, mean arterial pressure; ASA, American Society of Anesthesiologists (scale for physical health).

* The inclusion of ASA class III is a protocol violation.

### 3.4 High FRI, multivariate analyses

The multivariate analyses confirmed that FRI > 4.0 became less common with age and that an association existed with longer food intolerance times. FRI > 4.0 was also independently associated with lower urine output despite receiving a larger amount of Ringer’s solution during the surgery (all associations, *p* < 0.001; Table 3, top).

**Table 3:** Multiple logistic and linear regression analysis of the relationship between the Fluid Retention Index (FRI) and the perioperative variables.

Fluid Retention Index (FRI)	Factors in multivariate analysis	Odds ratio (95% CI)	*P*-value
FRI > 4.0(Dehydration)	Age (year)	0.94 (0.91–0.96)	<0.001
	Food intolerance (days)	1.22 (1.09–1.36)	<0.001
	Urine output (L)	0.102 (0.026–0.391)	<0.001
	Infused Ringer (L)	2.01(1.34–3.02)	<0.001
	Postoperative P-glucose (mmol/L)	0.72 (0.55–0.94)	0.02
FRI continuous scale 1–6	Extubation time (min)	1.011 (1.077–1.015)	<0.001
	MAP before induction (mmHg)	0.992 (0.984–0.999)	0.024
	Infused Ringer (L)	1.41 (1.28–1.55)	<0.001
	Urine output (L)	0.32 (0.25–0.41)	<0.001
	PACU time (min)	0.996 (0.992–0.999)	0.006
	MAP ratio surgery/baseline	1.76 (1.00–3.10)	0.048

The variables are presented in the order they were included in the stepwise regression.

MAP, mean arterial pressure; PACU, postoperative care unit.

When FRI was expressed on a continuous scale, multivariate analysis also identified that higher FRI was associated with lower MAP at baseline while the decrease during surgery was smaller (Table 3, bottom).

### 3.5 Postoperative AKI

**Figure 3:**
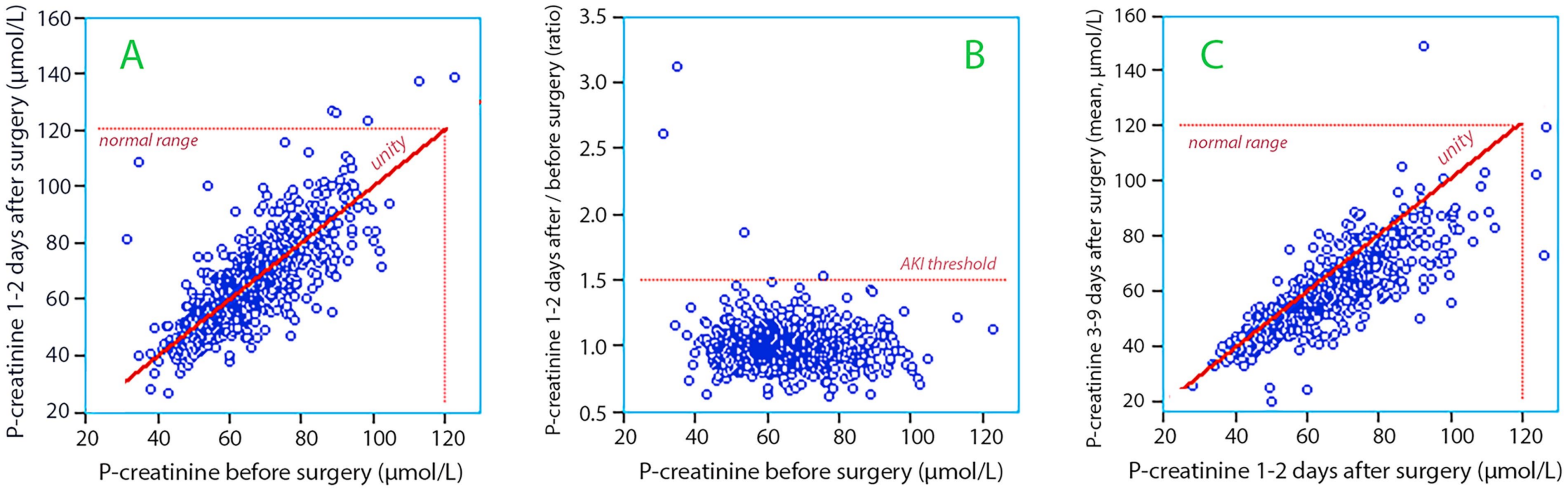
**(A)** The plasma creatinine concentration measured before surgery *versus* the concentration measured 1–2 days postoperatively. **(B)** Same data as in subplot A but the postoperative concentration is expressed as a ratio. Three of the four patients who fulfilled the criterion for Stage 1 of Acute Kidney Injury (AKI) had low plasma creatinine before the surgery (31, 34, and 54 μmol/L). **(C)** The plasma creatinine concentration measured 1–2 days after surgery *versus* the mean values of all measurements made 3–9 days postoperatively.

An increase of the plasma creatinine concentration of ≥50% from the preoperative value occurred in four patients (0.5%) on the first or second day following surgery (Figure 3A). Two of them had Stage 1 AKI, one had Stage 2, and one had Stage 3 AKI. However, three out of these four had very low preoperative levels (Figure 3B) and none of the increases surpassed the normal range (<120 μmol/L).

Five additional patients developed an increase in plasma creatinine ≥ 26.5 μmol/L (0.5%) which also classified them as AKI. Two of these patients marginally surpassed the normal range at 122 and 126 μmol/L.

All AKI patients belonged to the FRI ≤ 4.0 group, but the difference from the patients in the FRI > 4.0 group was still not statistically significant (chi-square *p* = 0.43 and *p* = 0.93 chi-square with Yates’s continuity correction; odds ratio 0.988, 95% CI 0.980–0.996). The 9 patients with AKI had a higher MAP before anesthesia was induced (102.7 ± 8.5 vs. 92.0 ± 11.8 mmHg; *p* < 0.01) and a greater decrease during the induction (−15.1 ± 6.2 vs. -5.1 ± 13.1 mmHg; *p* < 0.04) but MAP during surgery did not differ significantly from the other patients.

Between Days 3 and 9, plasma creatinine levels decreased in most patients (Figure 3C). During this period, 0.4% had elevations in plasma creatinine ≥ 50%. Only one patient was discharged with a plasma creatinine >120 μmol/L.

### 3.6 Outcomes, multivariate analyses

Binary multivariate regression confirmed that postoperative AKI was more likely in patients with a high MAP at baseline. An increased risk of AKI was also found in those who developed postoperative respiratory infection.

The other analyses per postoperative complication shown in Table 4 further support the association between FRI and food intolerance, paralytic ileus, and postoperative fever.

**Table 4:** Multiple linear and logistic regression analysis of the relationship between the demographic and surgical variables shown in Table 1 and the studied outcomes.

Morbidity	Factors in multivariate analysis	Relative risk (95% CI)	*P*-value
AKI (0/1)	MAP baseline (mmHg)	1.077 (1.020–1.139)	0.008
	Respiratory infection (0/1)	4.57 (1.19–17.59)	0.027
Paralytic ileus (days)	Infused Ringer (per 1 L)	2.25 (1.99–2.54)	<0.001
	BMI (per kg/m^2^)	0.94 (0.92–0.97)	<0.001
	FRI (continuous value)	1.16 (1.08–1.25)	<0.001
	Age (per year)	1.013 (1.006–1.020)	<0.001
	MAP surgery (mmHg)	1.018 (1.052–1.026)	<0.001
	ASA class (per grade)	1.38 (1.052–1.026)	<0.001
	Blood loss (per 100 mL)	0.90 (0.84–0.97)	<0.004
Food intolerance (days)	Infused Ringer (per 1 L)	3.99 (3.08–5.16)	<0.001
	BMI (per kg/m^2^)	0.91 (0.87–0.95)	<0.001
	FRI (continuous value)	1.34 (1.18–1.52)	<0.001
	Age (per year)	1.023 (1.011–1.035)	<0.001
	MAP surgery (mmHg)	1.032 (1.018–1.047)	<0.001
	ASA class (per grade)	2.07 (1.51–2.82)	<0.001
	Blood loss (per 100 mL)	0.79 (0.70–0.90)	<0.001
Respiratory infection (0/1)	Operating time (min)	1.003 (1.001–1.005)	0.022
	Pulmonary surgery (0/1)	7.51 (4.16–13.58)	<0.001
	Food intolerance (days)	1.126 (1.029–1.232)	0.01
	Age (year)	1.037 (1.020–1.056)	<0.001
	Urine flow (mL/min)	0.86 (0.77–0.96)	0.006
	GI surgery (0/1)	2.23 (1.22–4.09)	0.009
Wound infection (0/1)	Laparoscopic / Open (0/1)	6.03 (3.37–10.78)	<0.001
	Operating time (min)	1.005 (1.001–1.009)	<0.001
	Orthopedic surgery (no/yes)	2.67 (1.33–5.37)	0.006
	Food intolerance (days)	1.24 (1.09–1.41)	<0.001
	Fever (0/1)	1.90 (1.09–3.32)	0.024
	Paralytic ileus (days)	0.74 (0.56–0.98)	0.035
Fever (0/1)	GI surgery (0/1)	0.18 (0.10–0.30)	<0.001
	Respiratory infection (0/1)	2.78 (1.87–4.14)	<0.001
	Paralytic ileus (days)	1.32 (1.10–1.57)	0.002
	Wound infection (0/1)	2.10 (1.22–3.60)	0.007
	FRI > 4.0 (0/1)	2.10 (1.10–4.00)	0.024

The variables are presented in the order they were included in the stepwise regression.

Respiratory infection and wound infection were both associated with lengthy surgery. Respiratory infection was most common after pulmonary and gastrointestinal surgery while wound infection occurred after open surgery and orthopedic surgery.

Fever occurred with both types of infections and but was also twice as common in patients with preoperative FRI > 4.0 compared to those having a lower FRI.

More frequent occurrence of high pain scores (>4.0) was statistically associated with fever [relative risk (RR), 1.16 (95% CI 1.05–1.29)].

Wound infection was the only outcome significantly associated with reoperation [RR 14.5 (95% CI 4.0–52.2)].

Pulmonary surgery [RR 23.0 (95% CI 5.38–98.3)] and advanced age [1.24 per year (1.15–1.33)] were strongly associated a postoperative admission to intensive care. A large surgical hemorrhage [1.65 per liter (1.15–1.33)] were also over-represented among those who were referred to intensive care.

## 4 Discussion

Concentrated urine (FRI > 4.0) prior to surgery was associated with increased incidence of fever and with low urine output despite greater need for crystalloid fluid. There was also prolonged postoperative duration of paralytic ileus and food intolerance. Blood loss was slightly greater than that in other patients, a finding previously noted (17), and hemorrhage of clinical importance (>500 mL) was more common. Multivariate analysis confirmed that young age, a longer food intolerance time, and lower urine output despite greater need for crystalloid fluid were significantly associated with concentrated urine, while FRI expressed on a continuous scale also identified an association with lower MAP at baseline. The analysis per postoperative complication shown in Table 4 further supports the association between FRI and food intolerance, paralytic ileus, and postoperative fever.

Concentrated urine was still rare (7%) and AKI had a much lower incidence (1%) than in previous studies of major surgery; this skewed distribution made the study underpowered to confirm the hypothesized correlation between concentrated urine and AKI (4, 5). High MAP at baseline appeared to be the main factor that increased the risk of AKI; having 100 mmHg instead of 75 mmHg before the surgery would statistically increase the risk of developing AKI by almost 30%. However, when interpreting this result, one must keep the very low incidence of AKI in mind.

Several variables affirmed that patients with a high FRI in were dehydrated. They exhibited a lower MAP prior to the initiation of anesthesia and needed more intravenous fluid during surgery, which still resulted in less urine being excreted. Interestingly, high FRI also seemed to offer certain advantages, including a milder hyperglycemic response to the surgery. Yet only 7% of the cohort exhibited concentrated urine, suggesting that hydration was usually adequate when surgery was initiated.

Overall, the hemodynamics was stable and the urine flow adequate. Only 2.8% of the patients showed hypotension during more than 2 recordings. Moreover, after adjusting for body weight, the urine flow rate was 20% higher than in our previous study conducted across four countries (5). MAP is known to be closely associated with urine output during surgery (22) and, therefore, the high urine flow might be due to that MAP during the surgery was only slightly lower than before anesthesia was induced.

These three variables (hypotensive events, low urine output, and low mean MAP) increase the risk of a postoperative elevation of plasma creatinine and of AKI (5, 8, 19, 20, 23, 24). Urine flow <0.5 mL/kg/h during >6 h even serves as an independent criterion for the diagnosis (7). The good urine flow, well maintained hemodynamics, and only brief events of hypotension probably contributed, together with the good preoperative hydration, to the low incidence of AKI in the present study.

Elevations of plasma creatinine of ≥ 50% varies greatly after major surgery, from 2% (24), 6% (5), 8% (13), 12% (25), 20% (26), and up to 59% (27). These occurrences bear a risk of higher long-term morbidity and mortality (8, 25, 28, 29). The diagnosis does not require that the elevation is sustained, but in one study 10% of the patients who developed Stage 1 AKI showed a decrease of the glomerular filtration rate by 30% at 3 months after surgery, while the incidence was only 2% in those without AKI (9).

The ultimate cause of postoperative AKI has not been established, but the syndrome is considered to be multifactorial. Decreased kidney perfusion is a frequently suspected cause in the perioperative setting (7). Current research focus on finding new biomarkers of renal injury (30) and avoidance of arterial hypotension (31). Established preventive measures include identification of risk factors (metabolic syndrome, hypertension etc.), avoidance of nephrotoxins, as well as hemodynamic and intravascular volume optimization (6–8, 32).

In a large cohort study by Myles et al. (33) the incidence fell from 8.6 to 5.0% when liberal rather than restrictive fluid therapy was applied, which shows that adequate fluid therapy is important. Marques et al. (34) reported that an intraoperative fluid administration rate of <5 mL/kg/h, which is considered to be adjacent to “restrictive,” was strongly and independently associated with the development of postoperative AKI in bladder cancer patients. Furrer et al. (35) also found a higher occurrence of AKI when less crystalloid fluid was administered during bladder cancer surgery, but both rates averaged <5 mL/kg/h. The amount of fluid administered in the present study was clearly higher and can be placed in-between the liberal and restrictive fluid programs used in the study by Myles et al. (33). With regard to the fluid balance, we believe that the fairly liberal intravenous fluid administration despite small hemorrhage volumes, and possibly the use of a colloid fluid, contributed to the low incidence of AKI in our study.

Ellis et al. (36) have reported that patients who were dehydrated or mildly dehydrated had an increased risk of postoperative AKI after kidney tumor surgery. However, surgery on the kidney might be a special case with regard to the development of AKI. On the other hand, our previous study involving 642 patients across four countries showed that increasingly concentrated urine before general non-cardiac surgery correlated with gradually increasing plasma creatinine up to 25% postoperatively (5). The lack of a statistically significant relationship between FRI and AKI in the present study differs from the previous result and could be due to the low incidence of both variables, which gave the statistical analysis very low power. Statistical links between FRI and other postoperative complications were found, but these complications had a much higher incidence than AKI (Table 2).

A recent finding is that the incidence of AKI after cardiac surgery, which is a high-risk procedure, decreased from 31.7 to 26.9% after a 30-h infusion of amino acids (37) which has recently been confirmed in a meta-analysis (38). Loading with amino acids has several effects on renal physiology, including a recruitment of a “renal function reserve” which increases the glomerular filtration rate in those having a normal kidney function (39). Another effect is that an amino acid mixture increases the diuresis (40). Certain amino acids, such as glycine, even induce osmotic diuresis even in relatively modest amounts (41).

Intravenous fluid is administered to elective surgical patients under the assumption that all of them arrive at the hospital with a similar fluid status, but this may not be the case. Measuring the urine concentration by using FRI, or an individual metabolic end product, may help the clinician to evaluate if the daily intake of water is adequate or not (2, 3). However, the preoperative hydration status has not yet been acknowledged to play a role in the development of postoperative complications, including AKI.

The FRI scale is a tool used quantify concentrated urine. This scale was initially designed to detect dehydration following sports activities (12), but it can also be employed to approximate regular water consumption. Higher water intake decreases the FRI value quite slowly (days) as the regulating hormone, vasopressin, then operates in a low and narrow range (2). In a volunteer study, an increase of the daily intake of water by 32% had not decreased the urine concentration by 15–20% until 1 week later (3). Therefore, FRI in the morning urine is a stable indicator of the 24-h intake of water, although the accuracy decreases transiently in spot urine after ingestion of water (42). The kidneys increase the FRI value much faster in response to surgical stimuli, such as arterial hypotension and peritoneal stretching, due to a more dramatic vasopressin response (18, 20).

The FRI scale has also been applied in other healthcare settings, and patients have typically showed higher values than found here. FRI > 4.0 occurred in 16% of patients admitted to acute geriatric care and correlated with a statistically higher mortality rate within 30 days (14). It should be emphasized that the FRI should be assessed just before surgery starts to be independent of surgical stress. In our compilation of eight surgical patient studies, the average FRI was 3.0 (5), which is 30% higher than observed in this research. Pre-surgical patients with concentrated urine exhibited a 20% lower stroke volume than others (43). Notably, an elevated FRI tends to delay gastrointestinal recuperation post-surgery (19). Yet, the most compelling correlation discovered in previous work is the one between FRI and postoperative creatinine (5, 16). The present study has, to our knowledge, the lowest reported incidence of postoperative AKI in any published major cohort of surgical patients.

Concentrated urine is of great interest as predictor postoperative complications because it can be manipulated before surgery. The hydration status can be graded by measuring urine creatinine, urine osmolality, or even the FRI on the preoperative visit to the hospital before surgery. Additional intake of water could then be prescribed to patients with strong renal water conservation, although this type of prevention has not yet been attempted. Original data from a diet study suggests that, in those presenting with high urine creatinine (>12 mmol/L), an increase of the daily water intake by 700 mL decreases the concentration by 25% or more within 4 days (3). This volume corresponds to one additional glass of water to every meal.

Crystalloid fluid is considered the standard of care for intravascular volume expansion to prevent or treat AKI. This rationale is based on lack of clear evidence that colloids are superior (7). However, there is suspicion that specific colloids, such as Voluven, may cause AKI (44). The use of Voluven^®^ in the present study was not part of the study protocol and was administered to nearly all patients (93%). This fluid is widely used in China where this study was performed. The low incidence of postoperative AKI we found contradicts the concern that Voluven^®^ jeopardizes renal safety, which has resulted in regulatory limitations of its use in Europe and the US. In addition, three meta-analyses (45–47), two randomized trials (48, 49), and one recent retrospective case–control study of 11,000 paired Chinese patients (50) report no association between the use of Voluven^®^ and postoperative AKI. If anything, our study suggest that Voluven prevents AKI rather than promoting it because the incidence is, to the best of our knowledge, the lowest ever reported. However, this proposal must be confirmed by a randomized trial.

### 4.1 Limitations

To our knowledge, this is the largest prospective study of AKI that has been performed. Interestingly, it shows that the occurrence of AKI can be very low despite lengthy surgery, and we have suggested factors that we believe contributed to this result. A few of the AKI diagnoses can even be questioned. Three patients who were diagnosed with AKI had very low plasma creatinine levels before the study but normal values for the cohort at the postoperative follow-up (Figure 3B). This opens the possibility that sample dilution caused erroneous starting values.

Preoperative plasma creatinine and C-reactive protein could be measured 1 day before the surgery or just before induction of anesthesia and postoperative sampling was performed in the first morning after the surgery (72%) or delayed to the second day (28%). However, only minor changes in plasma creatinine occur between these days; elevations are even firmly established already within 6 h after the end of surgery (19) and decreases take place quite slowly, as shown in the present study.

The observational design of the study makes conclusions about mechanistic effects debatable. The statistical analyses demonstrate associations, which interpretation can cause dilemmas. For example, patients who received larger volumes of fluid may have been perceived as dehydrated by the anesthesiology team. Therefore, complications during the postoperative follow-up in patients with high FRI could potentially be the result of the larger amount of crystalloid fluid given to them (51). However, in this case, the multivariate analysis identified both FRI and the amount of infused fluid as being independent predictors of the food intolerance time (Table 4).

The length of hospital stay surpassed what is common in European and American hospitals but reflect local practices and the predominance of lengthy cancer surgery.

Surgery was lengthy but ASA Class III patients and those with kidney failure avoided, which limits generalizability. However, the study still comprised patients with hypertension and the metabolic syndrome, which are known risk groups for postoperative AKI (6–8, 35).

## 5 Conclusion

Concentrated urine compatible with low habitual intake of was uncommon (7%) before 921 surgical operations averaging 2.9 h in duration. Patients with concentrated urine were more likely to have low urine output during surgery despite larger volumes of administered intravenous fluid. They also had a longer period of food intolerance after the surgery and a higher occurrence of fever. The incidence of AKI was very low (1%), which we credited to the adequate preoperative hydration, maintenance of normal arterial pressure during surgery, and proper urine flow. The extremely low incidences of both concentrated urine and AKI rendered the study underpowered to demonstrate an association between these two variables.

## Data Availability

The original contributions presented in the study are included in the article/Supplementary material, further inquiries can be directed to the corresponding author.

## Supplementary material

SUPPLEMENTARY FILE 1 Surgical details on the subgroups (Table S1) and their outcomes (Table S2).

SUPPLEMENTARY FILE 2 All blood and urine chemistry (Page 1), arterial pressures (Page 2), and postoperative complications (Page 3).
