# Supplementary material for: Effects of concentrated urine on complications and plasma creatinine: a prospective study in elective non-cardiac surgery patients
Source: Front Med (Lausanne). 2025 Nov 17;12:1662177. doi: 10.3389/fmed.2025.1662177 (PMC12665721; doi:10.3389/fmed.2025.1662177)
Supplement: SUPPLEMENTARY FILE 1 — Surgical details on the subgroups (Table S1) and their outcomes (Table S2). [file Table_1.DOCX]

**Types of surgery**

The gastrointestinal surgeries involved the removal of colonic, rectal, and gastric cancer. The gynecological procedures encompassed hysterectomies and myomectomies. Orthopedic surgeries included hip and knee replacements, as well as neck and back surgeries. The liver and gallbladder surgeries were performed to extract cancer. The pulmonary operations were thoracoscopy-assisted lobectomies.

**Table S1**

**Basic data on the patient cohorts and selected data on the surgeries.** The statistical tests used were one-way ANOVA followed by the Scheffé post-hoc test, the Kruskal-Wallis test followed by the pairwise *post hoc* test in SPSS version 28.0.0 for Mac (IBM Corp., Armonk, NY). For categorical data, the chi-square test was used, with squared z-values determining statistical differences between sub-groups.

| Variable | Gastro-  intestinal | (2)  Gyneco-  logy | (3)  Orthopedic  surgery | (4)  Liver and  gallbladder | (5)  Pulmonary  surgery | P-value |
| --- | --- | --- | --- | --- | --- | --- |
| N | 476 | 97 | 99 | 66 | 183 | - |
| Females (%) | 34 | 100 | 52 | 46 | 50 | 0.001; 1<2-5, 2>1, 3-5 |
| Age (yr) | 63 ± 11 | 46 ±10 | 64 ± 11 | 60 ± 13 | 61 ± 10 | 0.001; 2 < 1, 3, 4 |
| Body weight (kg) | 60 ± 9 | 60 ± 8 | 63 ± 11 | 61 ± 11 | 61 ± 8 | 0.19 |
| BMI (kg/m2)  BMI < 25 | 22.4 ± 2.6  16.2 | 23.6 ± 3.0  25.8 | 23.7 ± 3.5  29.3 | 22.7 ± 3.0  18.2 | 22.9 ± 2.6  18.6 | 0.001; 1 < 2, 3  0.02; 3 differs |
| ASA class 1/2/3 (%) | 73 /24 / 3 | 99 / 1 / 0 | 82 / 18 / 0 | 86 / 11 / 3 | 75 / 25 / 0 | 0.001; 1 few ASA1 |
| FRI (mean score) | 2.4 ± 1.2 | 2.2 ± 1.1 | 2.1 ± 1.0 | 2.1 ± 1.0 | 2.2 ± 1.0 | 0.004; 1 > 3 |
| Ringer (mL) | 2300 ± 625 | 1176 ± 305 | 1539 ± 501 | 2297 ± 911 | 1791 ± 596 | 0.001, all differ |
| Time (min)  Surgery  Extubation  PACU | 197 ± 68  34 ± 23  72 ± 30 | 98 ± 37  23 ± 11  57 ± 19 | 134 ± 61  26 ± 14  69 ± 32 | 198 ± 104  34 ± 18  81 ± 32 | 162 ± 74  30 ± 17  71 ± 24 | 0.001; not 1 vs 4  0.001; 1 > 2, 3, 5  0.001; 2 < 4, 5 |
| Open surgery (%) | 44 | 17 | 100 | 21 | 3 | 0.001; all differ |
| Blood loss (mL) | 100 (50-120) | 20 (20-50) | 100 (73-230) | 200 (100-300) | 50 (30-100) | 0.001; all differ |
| MAP (mmHg)  Baseline  During surgery  Ratio | 91 ± 12  88 ± 10  0.98 ± 0.16 | 92 ± 10  93 ± 9  1.02 ± 0.12 | 97 ± 12  84 ± 8  0.87 ± 0.12 | 94 ± 14  86 ± 9  0.92 ± 0.13 | 91 ± 10  83 ± 8  0.92 ± 0.11 | 0.001; 3 > 1, 2, 5  0.001; 1 > 3, 5  2 > 1, 3, 4, 5  0.001; 1, 2 > 3-5 |
| Urine output  Volume (mL)  Flow (mL/min) | 400 (242-600)  1.6 (0.9-2.4) | 150 (50-300)  0.9 (0.5-2.1) | 300 (200-500)  1.8 (1.0-2.6) | 375 (200-500)  1.2 (0.9-1.8) | 350 (200-600)  1.6 (0.9-2.6) | 0.001; 2 < 3-5, 3 < 4  0.001; 2 <3, 5; 3 < 4 |

BMI, body mass index. FRI, Fluid Retention Index. MAP, mean arterial pressure. PACU, postoperative care unit.

ASA, American Society of Anesthesiologists (scale for physical health).

**Table S2.**

**Outcome measures.**

The statistical tests are the same as for Table S2.

| Variable | Gastro-  intestinal | (2)  Gyneco-  logy | (3)  Orthopedic  surgery | (4)  Liver and  gallbladder | (5)  Pulmonary  surgery | Statistics |
| --- | --- | --- | --- | --- | --- | --- |
| N | 476 | 97 | 99 | 66 | 183 |  |
| FRI > 4.0 (%) | 9.9 | 7.2 | 4.0 | 3.0 | 2.2 | 0.001; 2, 5 differ |
| Ringer > 3 L (%) | 8.2 | 0 | 2.0 | 19.7 | 2.2 | 0.001; 2,4,5 differ |
| MAP < 60 mmHg  Incidence (%)  Time/case (min) | 15  8 | 1  5 | 12  10 | 8  31 | 19  12 | 0.001; 2 < others  0.001; 2 < 1, 5 |
| C-reactive protein (µg/L)  Baseline  Postoperative | 1.7 (0.7-4.7)  43 (28-64) | 0.5 (0.3-1.3)  61 (46-107) | 1.3 (0.5-4.3) 53 (34-99) | 1.0 (0.4-2.3)  43 (29-62) | 0.8 (0.4-1.7)  95 (51-152) | 0.001; 2 < 1, 3, 4  0.001; 5 > 1, 3, 4; 3 > 1, 4 |
| P-creatinine (µmol/L)  Baseline  Postoperative  Change (%)  Increase >25% (%)  Increase > 50% /%) | 68.2 ± 13.5  68.9 ± 15.6  +1.2 ±17.1  3.7  0.2 | 54.5 ± 7.6  56.3 ± 5.4  +4.1 ± 12.8  0  0 | 62.8 ± 12.2  63.3 ±13.8  +1.7 ± 12.6  4.4  1.1 | 66.4 ± 13.5  64.8 ±15.1  –1.2 ± 0.26  6.1  3.0 | 65.5 ± 11.6  64.3 ±13.7 11.6 ± 14.4  2.8  0 | 0.0001; 1> 2,3,2 < 4,5  0.023; 1 > 3, 5  0.24  0.78  0.04; 4 > 1, 2, 5 |
| Paralytic ileus (day) | 2.4 ± 1.2 | 0.5 ± 0.6 | 0.3 ± 0.4 | 1.5 ± 1.0 | 0.4 ± 0.4 | 0.001; 1 > 2-5, 4 > 2, 3, 5 |
| Food intolerance (day) | 4.2 ± 2.1 | 0.7 ± 1.0 | 0.3 ± 0.6 | 2.3 ± 1.5 | 0.6 ± 0.9 | 0.001; 1 > 2-5, 4 > 2, 3, 5 |
| Infection (%)  Respiratory  Wound  Fever | 2  12  11 | 4  0  22 | 5  27  21 | 14  5  27 | 34  4  28 | 0.001; 4, 5 differ  0.001; 2, 3, 5 differ  0.001; 1 differs |
| VAS > 4 (%) | 23 | 7 | 34 | 27 | 33 | 0.001; 2, 3,5 differs |
| Hospital stay (days) | 11.2 ± 3.7 | 5.5 ± 1.7 | 7.8 ± 4.2 | 8.8 ± 2.9 | 6.6 ± 4.5 | 0.0001; 1 > 2-5  2 < 3,4; 4 > 5 |
| Intensive care (%) | 2.3 | 0.0 | 4.0 | 1.5 | 4.4 | 0.19 |

FRI, Fluid Retention Index. MAP, mean arterial pressure. PACU, postoperative care unit.

ASA, American Society of Anesthesiologists (scale for physical health). VAS, visual analogue scale.
